# High prevalence of cardiovascular risk factors in children and adolescents with Williams-Beuren syndrome

**DOI:** 10.1186/s12887-015-0445-1

**Published:** 2015-09-17

**Authors:** Daiji Takeuchi, Michiko Furutani, Yuriko Harada, Yoshiyuki Furutani, Kei Inai, Toshio Nakanishi, Rumiko Matsuoka

**Affiliations:** Department of Pediatric Cardiology, Tokyo Women’s Medical University, 8-1 Kawada-cho, Shinjuku-ku, Tokyo 162-8666 Japan; The International Research and Educational Institute for Integrated Medical Sciences (IREIIMS), Tokyo Women’s Medical University, 8-1 Kawada-cho, Shinjuku-ku, Tokyo 162-8666 Japan; International Center for Molecular, Cellular, and Immunological Research (IMCIR), Tokyo Women’s Medical University, 8-1 Kawada-cho, Shinjuku-ku, Tokyo 162-8666 Japan

**Keywords:** Williams-Beuren syndrome, Cardiovascular risk, Child, Adolescent, Elastin

## Abstract

**Background:**

A high incidence of cardiovascular (CV) risk factors has been reported in adults with Williams-Beuren syndrome (WS). However, the prevalence of these factors in children and adolescents with WS is unknown. Therefore, the purpose of this study was to evaluate the prevalence of CV risk factors in these patients.

**Methods:**

Thirty-two WS patients aged <18 years were enrolled in the study. Oxidized low-density lipoprotein levels (*n* = 32), oral glucose tolerance test results (*n* = 20), plasma renin and aldosterone levels (*n* = 31), 24-h ambulatory blood pressure (ABP; *n* = 24), carotid artery intima-media thickness (IMT; *n* = 15), and brachial artery flow-mediated dilatation (FMD; *n* = 15) were measured and analyzed.

**Results:**

The lipid profile revealed hypercholesterolemia in 22 % and elevated oxidized low-density lipoprotein levels in 94 % of the patients. Glucose metabolism abnormalities were found in 70 % of the patients. Insulin resistance was observed in 40 % of the patients. High plasma renin and aldosterone levels were detected in 45 and 39 % of the patients, respectively. A mean systolic blood pressure above the 90th percentile was noted in 29 % of patients. High IMT (>0.65 mm) and low FMD (<9 %) were detected in 80 and 73 % of patients, respectively.

**Conclusion:**

In patients with WS, CV risk factors are frequently present from childhood. In children with WS, screening tests for the early detection of CV risk factors and long-term follow-up are required to determine whether long-term exposure to these factors increases the risk for CV events in adulthood.

## Background

Williams-Beuren syndrome (WS) was first described in 1961 by Williams et al. [1]. They reported four patients with supravalvular aortic stenosis (SVAS), mental retardation, and characteristic facial features. At present, WS is recognized as a congenital developmental disorder involving the connective tissue and central nervous system, and it is accompanied by various characteristic facial features. The syndrome is characterized by growth delay, mental retardation with typical neurobehavioral profile [2], cardiovascular (CV) abnormalities, hypertension, hypercholesterolemia, hypothyroidism, and occasional infantile hypercalcemia. In 1993, Ewart et al. [3] reported that haploinsufficiency of multiple genes, including microdeletions of the elastin gene locus at *7q11.23*, contribute to the phenotypic features of WS. Loss of elastin function is responsible for the associated CV abnormalities [4]. In adult WS patients, CV risk factors such as diabetes mellitus, hypertension, and hyperlipidemia are frequently present [5]. The prevalence of these risk factors among children with WS remains unknown; however, we hypothesize that they are present from a young age. A high prevalence of CV risk factors in childhood would likely contribute to CV events during adulthood. The purpose of this study was to evaluate the incidence of such factors, including hypercholesterolemia, impaired glucose tolerance, high blood pressure, renin-angiotensin-aldosterone (RAA) activation, intima-media thickness (IMT) of the carotid artery, and endothelial dysfunction in children and adolescents with WS; this is an important step in the prevention of future CV events in WS patients.

## Methods

**Fig. 1:**
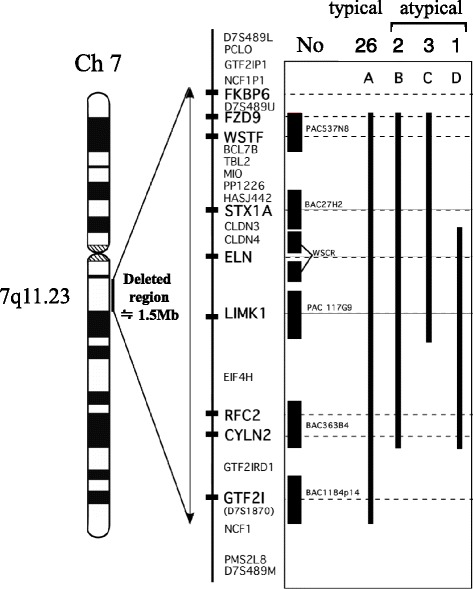
The chromosome 7q11.23 microdeletion was associated with the Williams-Beuren syndrome phenotypes in all subjects. Overall, 26 of 32 patients (82 %) showed the typical deletion responsible for Williams-Beuren syndrome (denoted as A), and 6 patients showed atypical deletions shorter than A (B, *n* = 2; C, *n* = 3; and D, *n* = 1)

We studied 32 WS patients aged <18 years. Fluorescence in situ hybridization (FISH) was performed using the following 6 probes: PAC537N8 (*WSTF and FZD9*), BAC27H2 (*STX1A*), WSCR (*ELN*), PAC 117G9 (*LIMK1*), BAC363B4 (*RFC2, CYLN2*), and BAC1184P14 (*GTF2I*), which included 8 genes for detecting microdeletions on chromosome *7q11.23*, as previously reported [6]. A typical deletion was revealed in 26 patients, and an atypical shorter deletion was discovered in 6 (Fig. 1). The following laboratory measurements were obtained: (1) a plasma lipid profile that included oxidized low-density lipoprotein (oxLDL) (*n* = 32) and lipoprotein-a (Lipo(a); *n* = 32); (2) glucose levels (via the oral glucose tolerance test (OGTT; *n* = 20); (3) plasma renin and aldosterone levels (*n* = 31); (4) 24-h ambulatory blood pressure (ABP; *n* = 24); (5) carotid artery IMT (*n* = 15); and (6) brachial artery flow-mediated dilatation (FMD; *n* = 15). All biochemical measurements in this study were performed using commercially available kits. The OxLDL and malondialdehyde-modified low-density lipoprotein (MDA-LDL) levels were measured (oxLDL, Kyowa Medics, Japan; *n* = 13 and MDA-LDL enzyme-linked immunosorbent assay, SRL, Japan; *n* = 19). A high oxLDL level was defined as oxLDL >7 U/L (normal: <6.9 U/L) or MDA-LDL >38.3 U/ L (normal: <38.2 U/L) in patients aged <18 years. Lipo(a) was also measured (SRL, Japan), and a high Lipo(a) level was defined as a level of >40 mg/ dL (normal: <39 mg/dL). The standard 2-h OGTT was performed in 20 patients with no history of diabetes, with the results classified according to the guidelines of the American Diabetes Association. Impaired fasting glucose level was defined as a glucose level of 100–125 mg/dL, and impaired glucose tolerance was defined as a plasma glucose level of 140–199 mg/dL 2 h after OGTT. Type 2 diabetes was defined as a fasting glucose level of ≥126 mg/dL or a 2-h plasma glucose level of >200 mg/dL [7]. The homeostasis model assessment insulin resistance index (HOMA-IR) was also measured, with insulin resistance defined as HOMA-IR >2. Impaired insulin secretion was defined as an insulinogenic index <0.4. Plasma levels of active renin were measured (PRA, SRL, Japan, respectively), and elevated renin levels were defined as levels > 3 pg/mL (normal range: 0.3–2.9 pg/mL). Plasma levels of aldosterone were measured (Aldosterone RIA kit II; Dinabot, Japan; *n* = 15 or SPAC-S Aldosterone Kit; TFB, Japan; *n* = 17). Elevated aldosterone levels were defined as levels >15 ng/dL (normal range: 2.2–14.9 ng/dL) and >159 pg/mL (normal range: 29.9–158 pg/mL) as measured using the SPAC-S aldosterone kit and RIA kit II, respectively. On the basis of ABP, high blood pressure (BP) was defined as a mean daytime BP above the 90th percentile corrected by sex, age, and height, as previously reported [8].

For IMT assessment, both common carotid arteries were measured in the longitudinal plane from the level of the clavicles to the carotid bifurcation, as previously reported [9]. Abnormal thickening was defined as IMT > 0.65 mm in individuals aged < 18 years [9–11]. FMD was measured in the right brachial artery after sublingual administration of nitroglycerin, in order to determine the extrinsic nitric oxide donor (nitroglycerin)-induced dilatation (NID), as previously reported [12]. Low FMD was defined as FMD < 9 %, and low NID was defined as FMD < 12 %. Ethical approval for this study was granted by the institutional review board of the Tokyo Women’s Medical University Hospital, Japan. Informed consent was obtained from the participants themselves or their parents in the case of children aged <16 years.

### Statistical analysis

Data are expressed as median (range) or mean ± standard deviation. Comparisons between the two groups were performed using the unpaired *t*-test or Mann–Whitney *U*-test. Pearson’s correlation coefficient was used to assess the associations between the two groups. Values were considered significantly different at *p* < 0.05. All analyses were performed using the JMP statistical software (version 11; SAS Institute, Cary, NC).

## Results

In the 32 WS patients, the median age of the subjects was 9.1 years (range, 1.3–17.9 years). The male: female ratio was 1:1.5, and the median height, body weight, and body mass index (BMI) were 122 cm (range, 78–147 cm), 25 kg (range, 6–41 kg) and 15.4 (range, 10.0–22.8), respectively. A BMI > 22 was observed in only 1 patient (3 %).

### Cardiovascular abnormalities

CV abnormalities were observed in 29 out of 32 patients (91 %). Among these, 24 patients had SVAS. The details of CV abnormalities are shown in Table 1. Five patients underwent surgical intervention for these abnormalities. Repair of the SVAS and ligation of patent ductus arteriosus had been performed previously in 4 and 1 patients, respectively. At the time of the study, no patient showed SVAS with an estimated pressure gradient of >50 mm Hg between the left ventricle and ascending aorta, as measured by echocardiography or cardiac catheterization. One patient had moyamoya disease.

**Table 1:** Summary of the cardiovascular abnormalities of the 32 patients

	Number
SVAS alone	7
SVAS with MVP	6
SVAS with pulmonary stenosis	5
SVAS with ventricular septal defect	2
SVAS with MVP and PAPVR	1
SVAS with MVP and pulmonary stenosis	1
SVAS with coarctation of the aorta	1
SVAS with a bicuspid aortic valve	1
MVP alone	3
MVP with patent ductus arteriosus	1
Pulmonary stenosis	1
None	3
	Total number = 32

SVAS: supravalvular aortic stenosis, MVP: mitral valve prolapse, PAPVR: partial anomalus pulmonary venous return

### Lipid profile

The results of lipid profile testing are shown in Table 2. Overall, 92 % (23/25) of the patients without hypercholesterolemia had high levels of oxLDL. The median level of total cholesterol, oxLDL, MDA-LDL, Lipo(a), high-density lipoprotein-cholesterol, and triglycerides were 166 mg/dL (range, 86–236 mg/dL),12.6 U/L (range, 7.5–46.6 U/L), 62.6 U/L (range, 28.6–116.0 U/L), 11 U/L (range, 3–99 U/L), 62 mg/ dL (range, 34–96 mg/dL), and 51 mg/dL (range, 33–116 mg/dL), respectively. There was no significant correlation between oxLDL levels and BMI. The results for the various parameters are summarized in Table 3.

**Table 2:** Summary of lipid profile test

	Number (Percentage)
*Lipid profile (n = 32)*	
Hypercholesterolemia	7 (22 %)
High oxidized LDL	30 (94 %)
High Lipo (a)	6 (19 %)
Hypertriglyceridemia	1 (3 %)
Low high-density lipoprotein cholesterol	2 (6 %)

LDL: low-density lipoprotein

**Table 3:** Summary of the results of various parameters

	Number (Percentage)
*Glucose tolerance (n = 20)*	
Impaired fasting glucose	2 (10 %)
Impaired glucose tolerance or DM by OGTT	14 (70 %)
Insulin resistance (HOMA-IR>2)	8 (40 %)
Impaired insulin secretion	3 (15 %)
*Renin-aldosterone system (n= 31)*	
Increased plasma renin level	14 (45 %)
Increased plasma aldosterone	12 (39 %)
*Ambulatory blood pressure monitoring (n=24)*	
High blood pressure >90 percentile	7 (29)
*Carotid artery ultrasound (n=15)*	
Increased IMT (>0.65 mm)	12 (80 %)
*Endothelial dysfunction (n=15)*	
FMD <9 %	11 (73 %)
NG-induced dilatation <12 %	2 (13 %)

OGTT: oral glucose tolerance test, DM: diabetes mellitus, HOMA-IR: homeostasis model assessment insulin resistance, IMT: intima-media thickness of the carotid artery, FMD: flow-mediated dilatation of the brachial artery, NG: nitroglycerin

### Impaired glucose metabolism

In this study, 14 patients demonstrated impaired glucose tolerance. Four and 10 of these patients were subsequently diagnosed with diabetes and impaired glucose tolerance, respectively. The median fasting blood sugar and insulin levels were 94 mg/dL (range, 80–103 mg/dL) and 7.5 μU/mL (range, 0.2–13.7 μU/mL), respectively. The insulinogenic index was 0.8 (range, 0.2–1.2) and the HOMA-IR was 1.8 (range, 0.03–3.1). The glycated hemoglobin level was <6.2 % in all patients, with a median level of 4.8 % (range, 4.6–5.0 %). A BMI of > 22 was observed in only 1 patient.

### Plasma renin and aldosterone activation

The median plasma renin level was 2.6 pg/mL (range, 0.3–13.0 pg/mL). The median plasma aldosterone level measured using the RIA kit II was 10.1 ng/dL (range, 5.2–22.9 ng/dL) and that measured using the SPAC-S kit was 165 pg/mL (range, 57.3–393 pg/mL).

### Ambulatory blood pressure monitoring

The median daytime BP in all subjects was 116 mm Hg (range, 100–131 mm Hg). The median daytime BP was 126 mm Hg (range, 120–147 mm Hg) and 109 mm Hg (range, 103–119 mm Hg) in the hypertensive and nonhypertensive groups, respectively. The BMI was not significantly different between the hypertensive and nonhypertensive groups (15.8 [14.4–18.0] vs. 16.1 [13.4–22.8], respectively).

### IMT of the carotid artery

**Fig. 2:**
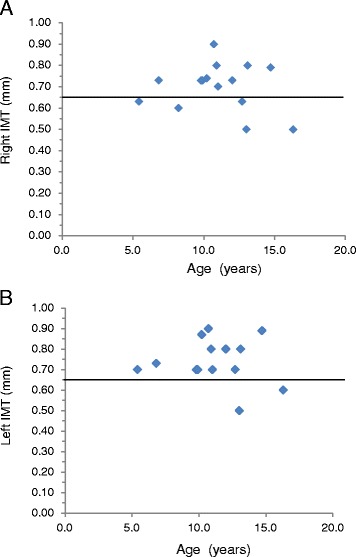
Relationship between intima-media thickness (IMT) of the carotid artery and age. **a** The right carotid artery and (**b**) the left carotid artery. Line indicates IMT = 0.65 mm, suggesting the upper limit in individuals aged below 18 years

The median IMT of the right and left carotid artery was 0.73 mm (range, 0.50–0.90 mm) and 0.71 mm (range, 0.50–0.90 mm), respectively. A high IMT in at least one carotid artery (>0.65 mm) was observed in 80 % of patients (12/15). The median IMT of the right carotid artery was 0.70 mm (range, 0.60–0.79 mm) and 0.73 mm (range, 0.50–0.90 mm) in the hypertensive and the nonhypertensive groups, respectively. The median IMT of the left carotid artery was 0.70 mm (range, 0.60–0.89 mm) and 0.71 mm (range, 0.50–0.90 mm) in the hypertensive and nonhypertensive groups, respectively. There were no significant differences in IMT between the hypertensive and nonhypertensive groups. There were also no significant correlations between age and IMT (left IMT: *R* = −0.02; right IMT: *R* = −0.04) or SVAS pressure gradients estimated using echocardiography and IMT (*R* = 0.3). The relationship between IMT of the carotid artery and age is summarized in Fig. 2a and 2b.

### FMD of the brachial artery

**Fig. 3:**
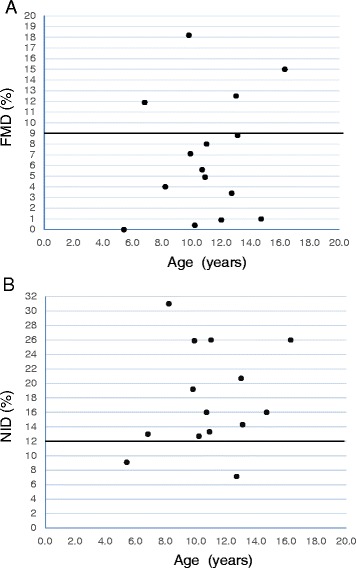
**a** Relationship between flow-mediated dilatation (FMD) of the brachial artery and age. Line indicates FMD = 9 %, suggesting the lower limit of FMD. **b** Relationship between extrinsic nitric oxide donor-induced dilatation (NID) of the brachial artery and age. Line indicates NID = 12 %, suggesting the lower limit of NID

The median FMD was 5.6 % (range, 0–18.2 %), and low FMD (<9 %) was detected in 73 % (11/15) of the patients. The median level of NID was 16 % (range, 7.1–25.9 %), and low NID (<12 %) was observed in 13 % (2/15) of the patients. The mean FMD was 4.3 % ± 3.2 % and 7.4 % ± 1.6 % in the hypertensive and nonhypertensive groups, respectively. The mean FMD between groups was not significantly different. There were also no significant correlations between BMI and FMD (*R* = 0.02), age and FMD (*R* = −0.13), or age and NID (*R* = −0.15). The relationship between FMD or NID of the brachial artery and age is summarized in Fig. 3a and 3b.

### Hypertension

There were no differences in any of the study measurements between the hypertensive and non-hypertensive groups.

### Deletions

Results of the *chromosome 7q11.23* microdeletion associated with the phenotypes in all WS patients are demonstrated in Fig. 1. Differences in the various parameters between the WS patients and the typical and atypical deletions are summarized in Table 4. Plasma renin and aldosterone levels measured by SPAC-S in patients with typical deletions (*n* = 26) were higher compared to those in patients with atypical deletions (*n* = 6). There were no significant differences in lipid profiles, impaired glucose metabolism, hypertension, IMT, and endothelial function between patients with typical deletions and those with atypical deletions.

**Table 4:**
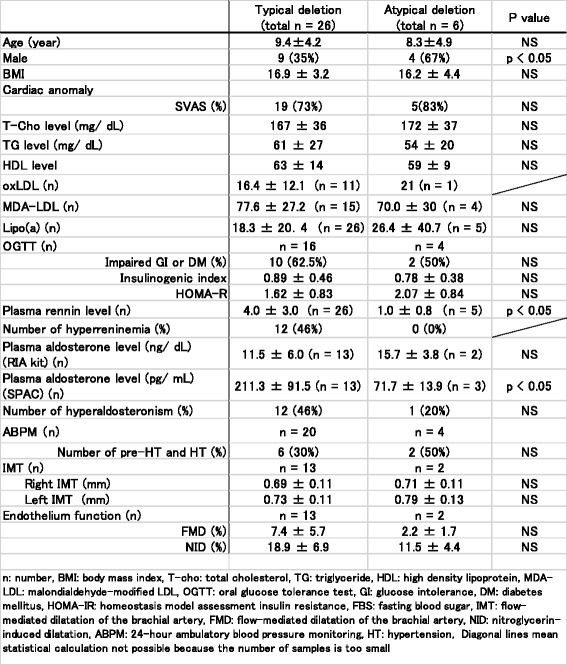
Differences in various clinical and laboratory parameters between the typical and atypical deletion groups

n: number, BMI: body mass index, T-cho: total cholesterol, TG : triglyceride, HDL: high density lipoprotein, MDA- LDL: malondialdehyde-modified, LDL, OGTT: oral glucose tolerance test, GI glucose intolerance, DM: diabetes mellitus, HOMA-R : homeostasis model assessment insulin resistance, FBS: fasting blood sugar, MT: flow- mediated

## Discussion

Decreased elastin function is known to be the underlying etiology for the cardiovascular lesions found in WS [10, 13–16]. In adult patients, a high prevalence of CV risk factors such as diabetes, hypertension, and hypercholesterolemia has been reported [5, 17–19]. However, the prevalence of such risk factors in children with WS has not been well studied. Risk stratification in children and adolescents is important, because a clear translation of CV risk factors in children into adulthood is expected to increase the incidence of future CV events [20]. As part of a holistic molecular genetic medicine approach [21] in this study, we evaluated the incidence of CV risk factors, including impaired glucose intolerance, hyperlipidemia, an activated renin-aldosterone system, endothelial dysfunction, and high IMT among children with WS.

### Hyperlipidemia and impaired glucose tolerance

In our study, 22 % of the patients had hypercholesterolemia. Interestingly, 92 % (22/25) of the patients without hypercholesterolemia demonstrated elevated levels of oxLDL. Oxidation of LDL is believed to be important in the development of early atherosclerosis, and oxLDL possesses several biologic properties that may promote atherogenesis [22, 23]. The OGTT results revealed that 70 % of the patients had impaired glucose tolerance or diabetes, which is consistent with the findings of previous studies on adult WS [5, 17–19, 24]. In this study, the high prevalence of elevated HOMA-IR revealed that hidden insulin resistance is present from childhood in WS patients. Both insulin resistance and an increased production of oxLDL may be associated with the progression of atherosclerosis in WS. Conversely, the elastin peptide that is derived from the degradation of elastin induces the oxidation of LDL by phagocytic cells and thereby promotes the initiation and progression of the atherosclerotic process [25]. Although obesity is strongly associated with hypercholesterolemia and insulin resistance in children as well as adults, only 1 patient in this study had a BMI of >22. The abnormal lipid or glucose metabolism in the rest of the children with WS was not associated with a high BMI. A close relationship between obesity and abnormal lipid/glucose metabolism is not possible with WS. This finding suggests that conditions other than obesity are involved in the high prevalence of CV risk factors in children with WS. Glucose dysregulation is caused by the hemizygosity of *syntaxin-1A*, a gene located in the WS chromosome region that is believed to be the prime candidate involved in insulin release [24].

### Activation of the RAA system and hypertension

Activation of the RAA system is associated with CV events and progression of arteriosclerosis [26]. In the current study, hypertension and activation of the RAA system were commonly found in the WS patients (Table 1), but there were no significant differences in the renin and aldosterone levels between the hypertensive and nonhypertensive groups. The etiology of hypertension among these patients appears to be multifactorial and potentially involves elastin haploinsufficiency, neutrophil cytosolic factor 1 hemizygosity [27], reduced nicotinamide adenosine dinucleotide phosphate-oxidase-mediated oxidative stress [28], an activated RAA system, and renovascular disease. In this study, patients with typical deletions exhibited a higher degree of RAA system activity than did patients with atypical deletions. However, the reason behind the high RAA system activity in the typical deletions group remains unclear. Whether microdeletion on *chromosome 7q11.23* leads to RAA system activation is also unclear. The precise role of the RAA system in WS should therefore be clarified in future studies.

### Brachial artery flow-mediated dilatation and carotid artery intima-media thickness

In this study, WS patients exhibited high carotid artery IMT, as previously reported [9]. An autopsy of a patient with WS revealed thickened medial tissue with elastic disorganization and a prominence of smooth muscle, not only in the arterial wall of the ascending aorta but also in the arteries of the lungs, kidneys, mesentery, and brain [6, 29]. Generalized arterial wall thickening with secondary lumen narrowing has also been observed in WS [10]. Therefore, this feature is considered a generalized elastin arteriopathy that presents in WS. Thickened medial tissue is thought to be primarily associated with high IMT in these patients; however, ultrasonography cannot differentiate between intimal atherosclerosis and medial hypertrophy.

Low FMD was also observed in the patients in this study. The current study is the first to demonstrate a high prevalence of impaired endothelial function associated with WS. FMD was considered an indicator of vascular endothelial function. Juonala et al. [12] reported that thickened IMT and low FMD are related to CV events. Even in children with diabetes mellitus, obesity, and hyperlipidemia, low FMD reflects impaired endothelial function from early childhood [30–34], and long-term exposure to such risk factors is associated with the development of atherosclerosis later in life [30]. The overall incidence of hypercholesterolemia and hypertension in Japanese school-age children has been reported to be 7 and 1 %, respectively [35], lower than the rates observed in our study. Although the number of patients enrolled in our study was small, the results strongly suggest that exposure to CV risk factors is present from childhood in patients with WS.

Diet and exercise intervention for obese children can reverse the pro-atherosclerotic inflammatory process and preserve vascular function [36]. Good familial support in order to avoid stress, which affects both mental and metabolic states, as well as the appropriate administration of drugs and supplements, may also be useful against the pro-atherosclerotic process in WS [16]. Minoxidil, glucocorticoids, retinoids, vitamin E and C, and matrix metalloproteinase inhibitors have the potential to upregulate elastin production or prevent its degradation [16, 25, 37–40]. Nutritional supplements such as β-aminopropionitrile (contained in certain legumes), dill extract, and tannic acid may also be effective in preventing elastin arteriopathy [16, 41, 42]. As part of a holistic approach for the management of patients with WS, we encourage patients to follow a traditional low-fat Japanese diet including rice, seaweed, wheat, barley, beans, fish, and vegetables and avoid other animal protein. We also recommend supplementation with vitamin C and E and good familial support. Two patients from a similar study experienced a decrease in IMT and an improvement in FMD as a result of this regimen [21].

## Conclusion

CV risk factors such as hypertension, impaired glucose tolerance, hyperlipidemia, and high IMT are highly prevalent in children with WS. Long-term exposure to these factors may accelerate the development of CV disease in adulthood. WS is a multisystem disorder that requires long-term follow-up, because WS is diagnosed during childhood in most cases. Health care supervisors and pediatricians, who care for children with WS need to be aware of these findings [43]. Screening tests for the early detection of CV risk factors such as abnormal lipid/glucose metabolism unrelated to obesity and long-term follow-up in WS patients could be helpful for preventing CV events in adulthood. Further studies are also needed to clarify the etiology of the various risk factors influencing CV events in these patients.

## Abbreviations

ABP: 24-h ambulatory blood pressure
BMI: Body mass index
BP: Blood pressure
CV: Cardiovascular
IMT: Intima-media thickness
FMD: Flow-mediated dilatation
HOMA-IR: Homeostasis model assessment insulin resistance index
Lipo(a): Lipoprotein-a
MDA-LDL: Malondialdehyde-modified LDL
NID: Nitric oxide donor (nitroglycerin)-induced dilatation
OGTT: Oral glucose tolerance test
oxLDL: Oxidized low-density lipoprotein
RAA: Renin-angiotensin-aldosterone
SVAS: Supravalvular aortic stenosis
WS: Williams-Beuren syndrome
